# Optically stimulated luminescence dosimeters for simultaneous measurement of point dose and dose-weighted LET in an adaptive proton therapy workflow

**DOI:** 10.3389/fonc.2023.1333039

**Published:** 2024-01-08

**Authors:** Mislav Bobić, Jeppe B. Christensen, Hoyeon Lee, Evangelia Choulilitsa, Katarzyna Czerska, Michele Togno, Sairos Safai, Eduardo G. Yukihara, Brian A. Winey, Antony J. Lomax, Harald Paganetti, Francesca Albertini, Konrad P. Nesteruk

**Affiliations:** ^1^ Department of Physics, ETH Zurich, Zurich, Switzerland; ^2^ Department of Radiation Oncology, Massachusetts General Hospital and Harvard Medical School, Boston, MA, United States; ^3^ Paul Scherrer Institute, Villigen, Switzerland

**Keywords:** OSLD, optically stimulated luminescence, proton therapy dosimetry, LET measurement, adaptive proton therapy, Monte Carlo, head and neck phantom, intensity-modulated proton therapy

## Abstract

**Purpose:**

To demonstrate the suitability of optically stimulated luminescence detectors (OSLDs) for accurate simultaneous measurement of the absolute point dose and dose-weighted linear energy transfer (LET_D_) in an anthropomorphic phantom for experimental validation of daily adaptive proton therapy.

**Methods:**

A clinically realistic intensity-modulated proton therapy (IMPT) treatment plan was created based on a CT of an anthropomorphic head-and-neck phantom made of tissue-equivalent material. The IMPT plan was optimized with three fields to deliver a uniform dose to the target volume covering the OSLDs. Different scenarios representing inter-fractional anatomical changes were created by modifying the phantom. An online adaptive proton therapy workflow was used to recover the daily dose distribution and account for the applied geometry changes. To validate the adaptive workflow, measurements were performed by irradiating Al_2_O_3_:C OSLDs inside the phantom. In addition to the measurements, retrospective Monte Carlo simulations were performed to compare the absolute dose and dose-averaged LET (LET_D_) delivered to the OSLDs.

**Results:**

The online adaptive proton therapy workflow was shown to recover significant degradation in dose conformity resulting from large anatomical and positioning deviations from the reference plan. The Monte Carlo simulations were in close agreement with the OSLD measurements, with an average relative error of 1.4% for doses and 3.2% for LET_D_. The use of OSLDs for LET determination allowed for a correction for the ionization quenched response.

**Conclusion:**

The OSLDs appear to be an excellent detector for simultaneously assessing dose and LET distributions in proton irradiation of an anthropomorphic phantom. The OSLDs can be cut to almost any size and shape, making them ideal for in-phantom measurements to probe the radiation quality and dose in a predefined region of interest. Although we have presented the results obtained in the experimental validation of an adaptive proton therapy workflow, the same approach can be generalized and used for a variety of clinical innovations and workflow developments that require accurate assessment of point dose and/or average LET.

## 1 Introduction

Clinical innovations and translational research in radiotherapy often require precise dose measurements in different phantoms to validate the expected dose distribution and its variation according to different irradiation conditions. In proton therapy, another important parameter is the linear energy transfer (LET). It is considered a surrogate for the relative biological effectiveness (RBE) (1), even though RBE does not necessarily scale linearly with LET (2, 3). Dosimetry, however, is challenged by the elevated LET at the spread-out Bragg peak (SOBP), which limits the use of many dosimeter types due to ionization quenching (4). Therefore, for accurate ion beam dosimetry, a dosimeter subject to quenching must also be able to assess the radiation quality, e.g. through an estimation of the average LET, in order to correct the measured dose for quenching. Besides the capability of assessing both dose and LET distributions simultaneously, the detector should only disturb the radiation field negligibly. One example application is the experimental validation of the dose and LET distributions in an online adaptive proton therapy workflow in an anthropomorphic phantom.

Passive luminescent detectors have been applied for simultaneous dose and LET determination in light ion beams for decades (5, 6). Among the available detector types, particularly the use of optically stimulated luminescence detectors (OSLDs) is attractive for in-phantom measurements due to the possibility of creating ultra-thin detectors (< 100 µm) that can be cut to arbitrary shapes (7). While previous studies have determined the dose and LET with OSLDs in proton beams under reference conditions, we demonstrate for the first time how these quantities can be measured in the mixed particle fields relevant to SOBPs to validate adaptive radiotherapy (8–10). Although the LET can be averaged in different ways, in our work we always refer to the dose-weighted LET (LET_D_) with contributions from protons only.

Adaptive radiotherapy refers to fractionated treatment delivery that takes into account changes in the patient’s anatomy during treatment. Geometric changes can occur within a fraction (intra-fractional) or between consecutive fractions (inter-fractional). The former is mostly due to respiratory motion, which particularly affects some tumor sites such as the abdomen and thorax. The latter can have various causes, such as weight gain or loss, tumor shrinkage, or even sinus filling. The goal of adaptation is to restore the original coverage of the target while sparing as much healthy tissue as possible. Proton therapy can particularly benefit from adaptation due to its well-defined range and sharp gradients. Daily variations in a patient’s geometry can affect the range of the protons, resulting in under-dosing of the target or over-dosing of organs at risk (OARs). Several adaptive proton therapy workflows have been proposed (11–30).

In this work, we report on the use of OSLDs for experimental testing of the adaptive workflow developed at the Paul Scherrer Institute (PSI) based on daily analytical plan recalculation according to the computed tomography (CT) of the day (21–24). To simulate realistic anatomical and positioning changes, we employed an anthropomorphic head-and-neck phantom previously developed at PSI. Our approach can be generalized and used in other experimental validations of proton therapy developments that require an accurate evaluation of point dose and/or LET.

## 2 Materials and methods

### 2.1 Anthropomorphic head-and-neck phantom

Dose delivery and measurements were conducted with PSI’s *Gantry 2* (31) using the anthropomorphic head-and-neck phantom. The phantom was developed at PSI in collaboration with CIRS (Computerized Imaging Reference Systems, Inc., Norfolk, USA) and used in a previous study to validate the daily adaptive proton therapy workflow developed at PSI (32). It is constructed from tissue-equivalent material and sliced into five sections along the coronal plane. The nasal cavities can be filled with mucus-equivalent material to mimic the effects of nasal congestion in head-and-neck patients. In addition, a fat layer can be applied to the neck area to simulate weight changes. Further details on the phantom can be found elsewhere (32).

**Figure 1:**
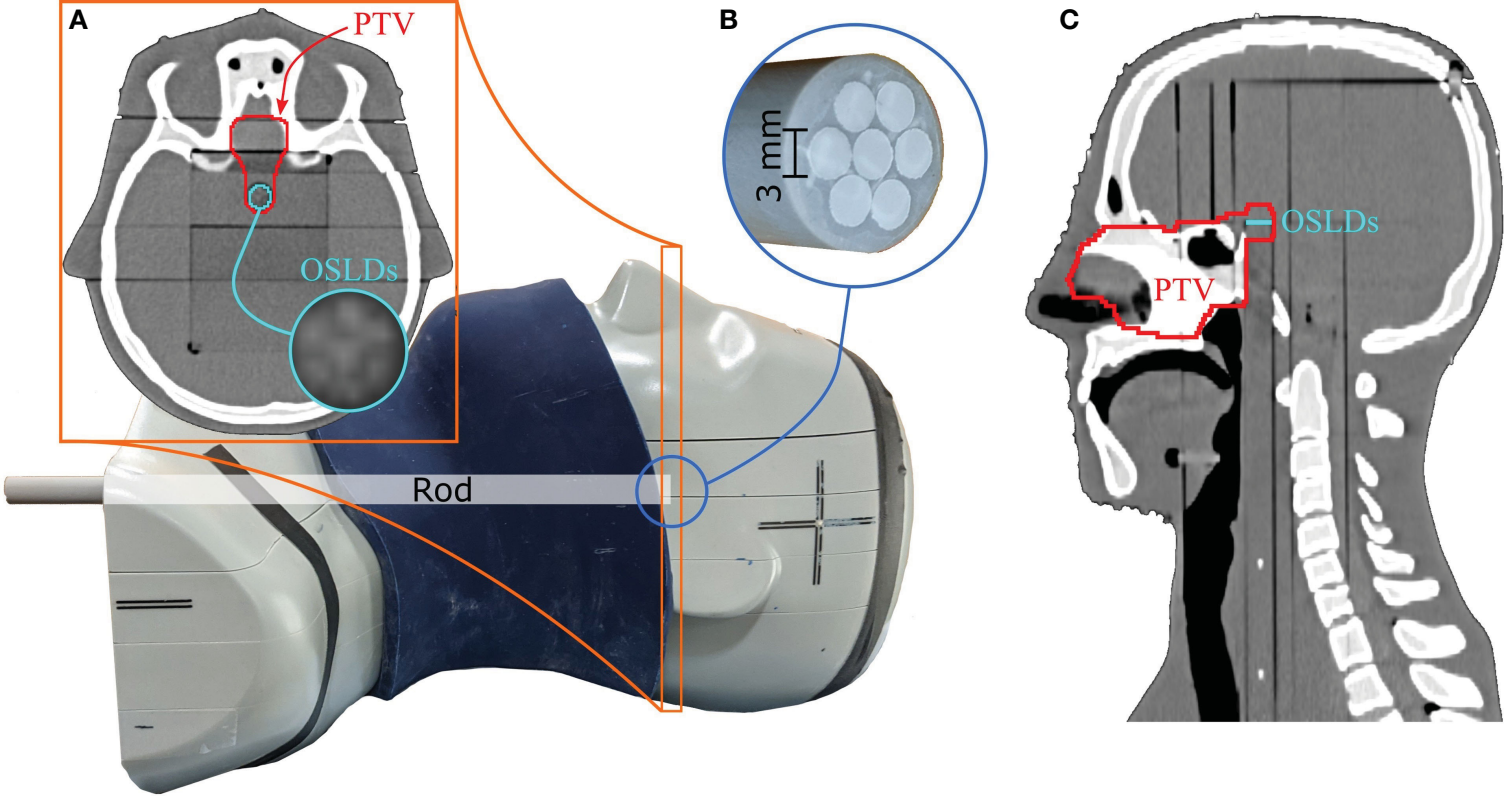
Anthropomorphic head-and-neck phantom used for measurements. The 1 cm fat layer is positioned around the phantom in this figure. The circular OSLD arrangement is visible on the axial CT slice in **(A)**. Seven 3-mm OSLDs are attached to the top of the tissue-equivalent rod, as seen in **(B)**. The PTV is extended posteriorly to include the OSLD contour, as seen in the axial and sagittal CT slices in **(A, C)**.

For our study, a reproducible position of the OSLDs in the phantom was required. To accomplish this, we positioned the OSLDs on top of a tissue-equivalent rod (see **Figure 1**) placed in the cylindrical channel drilled through the phantom. Sliding the rod to the end of the channel ensured consistent positioning across all measured fractions. Seven 3-mm diameter OSLDs were fixed in a circular manner (see section 2.4), with one positioned at the center of the rod as seen in **Figure 1B**. **Figure 1A** shows the axial slice of the CT with the OSLD arrangement visible on the scan. To simulate anatomical changes, we applied different configurations of the nasal cavity fillings as well as the 1 cm fat layer around the neck area.

### 2.2 Treatment planning and the DAPT workflow

The nominal treatment plan was created on the reference (planning) CT imaged with the PSI *Gantry 2* in-room CT scanner (33). We fixed the head-and-neck phantom with a thermoplastic mesh mask and a moulage to ensure consistent positioning between the reference CT and the consequent fraction images. Various regions of interest (ROIs), including OARs and a clinical target volume (CTV), were delineated by an experienced radiation oncologist. In addition, we extended the superior part of the CTV posteriorly to cover the OSLDs, as shown in **Figure 1**. A planning target volume (PTV) was then created by isotropically expanding the CTV by 2 mm.

Treatment planning was performed using PSI’s in-house treatment planning system, FIonA. The CTV received a prescription of 2.0 Gy(RBE) per fraction, assuming a constant RBE of 1.1 (physical dose = 1.82 Gy). Three anterior fields were introduced to deliver a combined uniform dose to the PTV with the following gantry angles: -20°, 0°, and 20°. The nominal plan was then optimized to the PTV with a range robustness setting of ± 3.0% and additional OAR objectives/constraints to fulfill the QUANTEC guidelines (34). The resulting IMPT plan served as the *reference plan* for the online plan adaptation.

Online plan adaptation was performed using PSI’s daily adaptive proton therapy (DAPT) workflow (12, 32). The *reference plan*, created on the reference CT, defines the beam geometries and ROI objectives/constraints to be used for daily dose re-optimization based on the anatomy of the day: first, daily imaging is performed with the same in-room CT scanner as for reference imaging, followed by rigid registration. The resulting vector field is used to propagate contours from the reference CT to the daily image. Once the daily ROIs are defined, the *daily plan* is created by optimizing the dose using the same set of objectives and constraints as in the *reference plan*. The *daily plan* is then delivered after clinical and physical quality assurance. More details on DAPT can be found elsewhere (32).

### 2.3 Treatment delivery and fractions

Two delivery methods were considered: the non-adaptive (NA) scenario and the daily adaptive (DAPT) scenario. In NA, the *reference plan* from the nominal/reference CT was delivered to the daily fraction image without any corrections. In DAPT, a *daily plan* was created and optimized based on the daily image using the DAPT workflow described above.

**Table 1** shows an overview of the delivered and measured fractions simulated by applying different combinations of positioning and anatomical changes compared to the reference CT. The nominal treatment plan was designed on the reference CT, which we refer to as the nominal positioning and nominal anatomy composition: no fat layer and nasal cavities filled with the mucus-equivalent material. Fraction 1 was a replication of the nominal/reference conditions, and only the NA scenario was measured for this fraction. For the remaining fractions, both NA and DAPT scenarios were delivered and measured. We used the same in-room CT scanner that was used for the reference CT to image the fractions.

**Table 1:** Overview of the delivered fractions.

Fraction #	Positioning	Anatomy	Delivery
Ref. CT	Nominal	Nominal (no fat layer, full nasal cavities)	
1	Nominal	Nominal	NA
2	Nominal	1 cm fat layer	NA, DAPT
3	1 cm lateral shift	Nominal	NA, DAPT
4	Nominal	½ nasal cavity fillings	NA, DAPT
5	Nominal	empty nasal cavity fillings	NA, DAPT

NA refers to the non-adaptive scenario, while DAPT refers to the daily adaptive scenario.

In fraction 2, we applied the 1 cm fat layer in the neck area as previously shown in **Figure 1**. This also affected the position of the phantom, as the fat layer induced both a spatial offset and an angular deviation compared to the reference. In fraction 3, we removed the fat layer and moved the phantom 1 cm laterally from its nominal position. In fraction 4, the phantom was in its nominal position, and we removed half of the nasal cavity fillings (starting from the most posterior section). In fraction 5, the phantom remained in its nominal position, and we removed the remaining half of the mucus-equivalent material, leaving the nasal cavities empty.

**Figure 2:**
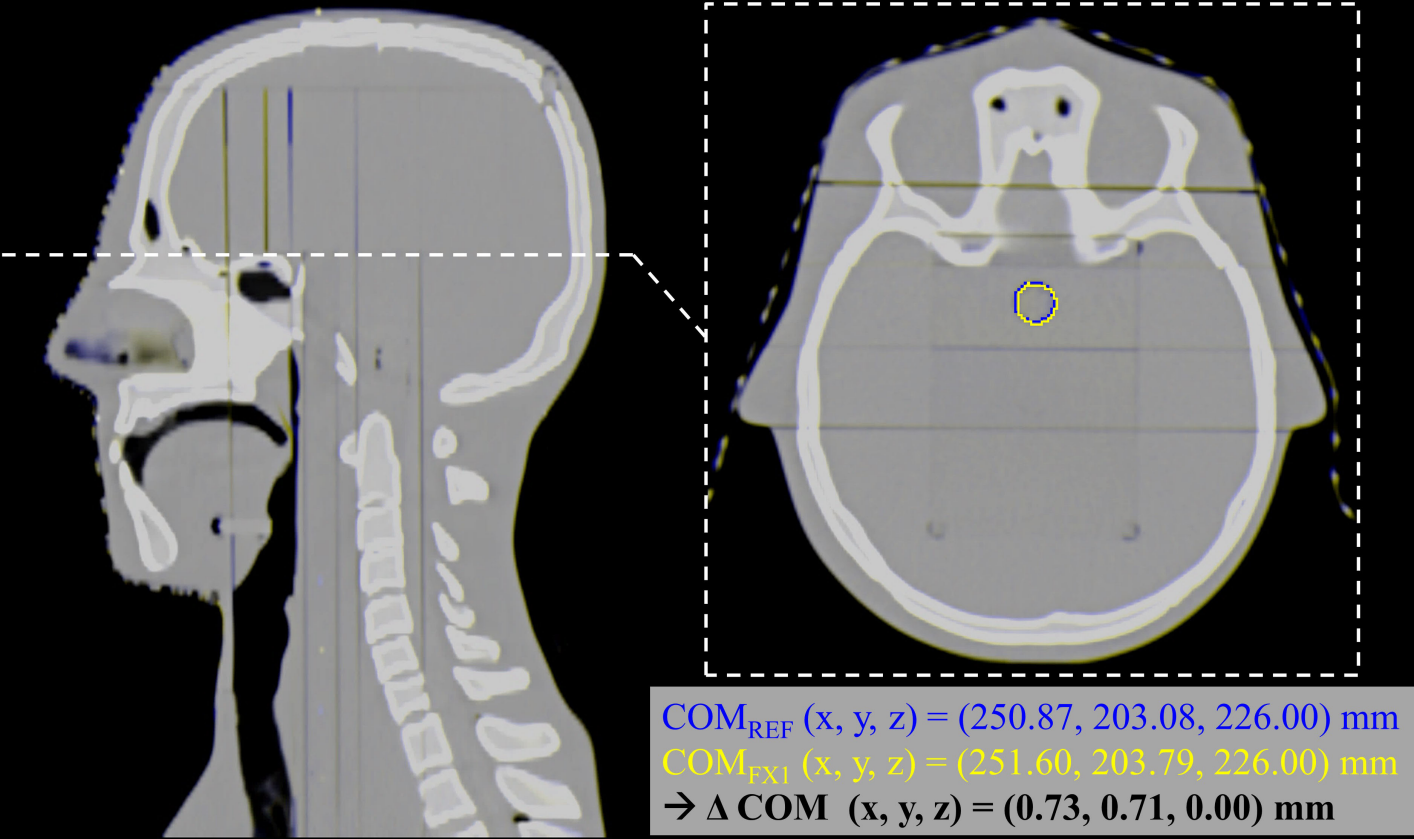
Estimation of the positioning reproducibility of the phantom and the OSLDs. Both the nominal/reference CT and fraction 1 CT are overlayed in complementary colors (blue and yellow, respectively), highlighting the positioning errors between the two images. On the axial slice, the OSLD contour is visible for both images, along with a calculation of the COM relative to the mutual CT origin. The difference between the two calculated COMs yields sub-millimeter accuracy in all three dimensions.

To confirm our positioning reproducibility, a quantitative measure is given in **Figure 2**. The figure overlays the nominal/reference CT and the CT of fraction 1 in complementary colors, blue and yellow, respectively (both at 50% transparency). The positioning errors can be seen by the blue/yellow shades throughout the image, especially visible at the edges and between the plates of the phantom. In addition, the OSLD contours for both CTs are visible on the axial slice, along with a calculation of the center of mass (COM) relative to the mutual CT origin. The difference between the two calculated COMs yields sub-millimeter accuracy in all three dimensions.

### 2.4 OSL detector preparation

All measurements were conducted with optically stimulated luminescence (OSL) Al_2_O_3_:C detectors. The 3-mm diameter OSLDs were cut from a single film made from < 38 μm Al_2_O_3_ grains mixed with a binder and a 75 μm polyester substrate as described in Ref (35). The OSLDs were optically bleached with a green light prior to irradiation. The OSLDs were placed inside the phantom as shown in **Figure 1**, which also served to shield the OSLDs from ambient light after irradiation. The OSLDs were fixed to the rod using a piece of tape sticking to the polymer side.

### 2.5 OSL readout and dosimetry

**Figure 3:**
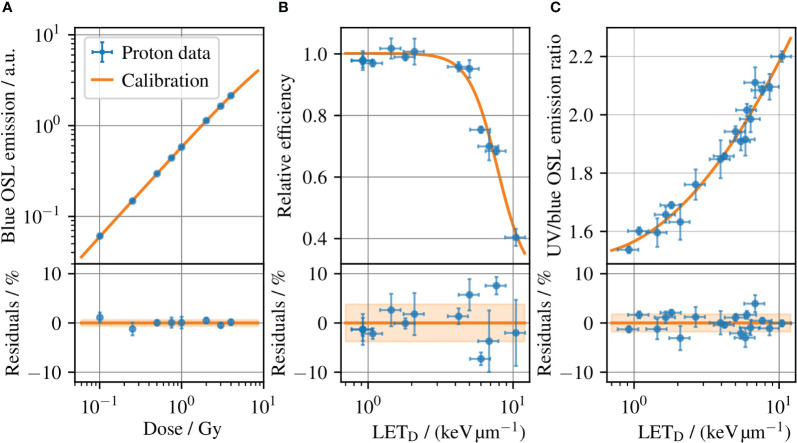
**(A)** The OSLD dose calibration for 240 MeV protons, which relates the blue OSL emission intensity to the dose measured with an ionization chamber. **(B)** Relative detector efficiency for the blue OSL emission for protons, where the emission band is subject to ionization quenching increasing with the LET. **(c)** Ratio of the UV and blue emission bands versus LET at reference conditions, i.e. the LET calibration. Error bars illustrate the (coverage factor *k=1*) uncertainties. The shaded bands in the residual plots outline the standard deviation of the residuals, which is 0.6%, 3.8%, and 1.8% for **(A–C)**, respectively.

To determine the dose and LET to each Al_2_O_3_:C OSLD, the detectors were read out using pulsed stimulation to separate the blue emission band from the UV emission (36). The OSLDs were read out in a commercial reader (Risø TL/OSL-DA-20, DTU Nutech, Denmark) in an automated sequence defined in Ref (37). Only the blue emission band was used for dosimetry through the dose calibration in **Figure 3A** obtained with 240 MeV protons, due to its dose linearity below 5 Gy relevant to this study along with a low fading-rate and dose-rate independence (37). To improve the dosimetry and reduce the effects of inter-sample differences, the OSLDs were subject to a reference irradiation with a known dose to normalize the signal (7). This enables a standard deviation of the residuals lower than 1% as seen in **Figure 3A**.

One challenge with OSLD dosimetry in light ion beams is its ionization-quenched response, in line with other solid-state detectors (38–41). The relative detector efficiency for the blue OSL emission band is shown in **Figure 3B** for protons, where the efficiency decreases with increasing LET. Unless corrected, the quenched response will lead to an underestimated dose for measurements in an SOBP. However, an LET correction can be derived from the OSL response itself through the ratio of the UV and blue emission bands. **Figure 3C** shows the ratio of the two OSL signals as a function of LET_D_, where the OSLDs were irradiated at different radiation qualities and the LET_D_ at each position assessed with Monte Carlo simulation methods. A detailed explanation of the data can be found in (7). The relationship between the UV and blue emission band ratios enables a determination of the LET_D_ for each OSLD readout. The use of the OSLD emission band ratio to determine LET_D_ has been demonstrated for protons, helium, and carbon ions up to 41.3 keV/μm in water. For ions heavier than carbon, the dense ionizations cause a signal saturation, and the emission band ratio is difficult to relate to the LET (10). The estimated LET_D_ can in turn be used to look up the relative detector efficiency in **Figure 3B** to correct ionization quenched dose in the OSLD. Hence, a single OSLD permits to determine the dose and average LET simultaneously (8, 10).

### 2.6 Monte Carlo dosimetry

In each delivered and measured fraction, both dose and LET_D_ were retrospectively simulated using MOQUI (42), an open-source GPU-based Monte Carlo code. For this purpose, we implemented the PSI *Gantry 2* beam model in MOQUI and cross-validated dose calculations in water with the treatment planning system FIonA.

MOQUI scores both dose and LET_D_ distributions on the CT grid separately for each treatment field. d*E*/d*x* was calculated by dividing a particle’s energy loss (d*E*) by the corresponding travel distance (d*x*). The ${\text{LET}}_{{\text{D}}_{\text{F}}}$ to each voxel ( ν ) with the density ( $\rho_{\nu }$ ) is scored as in Ref (43) with a water density $\rho_{water}$ of $1\text{ g}/{\text{cm}}^{3}$ and weighted by the energy deposition:


$${\text{LET}}_{{\text{D}}_{\text{F}}}(v)=\frac{\sum \text{d}E_{v}\cdot {\left(\frac{\text{d}E}{\text{d}x}\right)}_{v}}{\rho_{v}\cdot \sum \text{d}E_{v}}\cdot \rho_{water}$$

The above equation calculates ${\text{LET}}_{{\text{D}}_{\text{F}}}$ distributions for each field F, which are then used together with the field doses D_F_ to calculate the total LET_D_:


$${\text{LET}}_{\text{D}}(v)=\frac{\sum_{\text{F}}{\text{LET}}_{{\text{D}}_{\text{F}}}(v)\times {\text{D}}_{\text{F}}(v)}{\sum_{\text{F}}{\text{D}}_{\text{F}}(v)}$$

The precision of the Monte Carlo dose calculation is related to the number of particles simulated. We simulated particles on the CT images of the phantom (i.e., based on its density and structures) until a statistical uncertainty threshold of 1% was reached for the dose in the target. The voxel-wise dose and LET_D_ distributions were then averaged over a single-slice OSLD contour created on the axial CT slice to compare the Monte Carlo dosimetry results with the measurements. We contoured this structure to encompass all seven OSLDs on the tissue-equivalent rod (see **Figure 1**).

## 3 Results

### 3.1 OSLD dosimetry and LET determination

**Figure 4:**
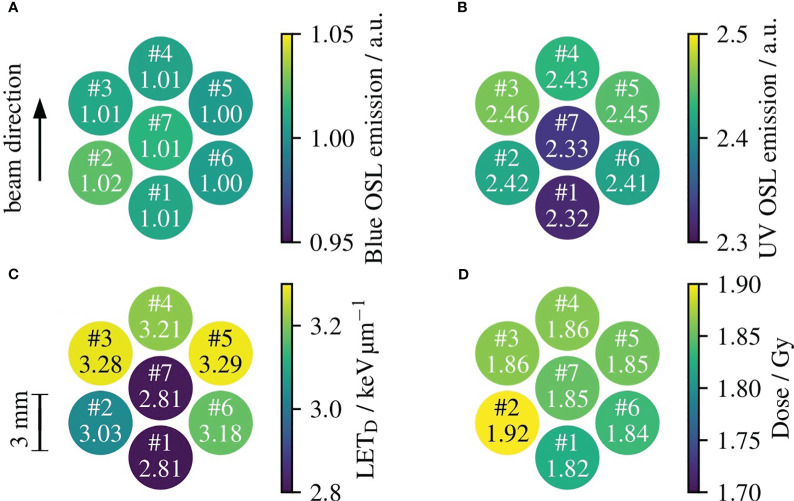
Example of the seven OSL detectors irradiated during the DAPT fraction 2. Each OSLD is annotated with its position number and the numerical value of the readout with units given in the color bar. **(A, B)** show the relative blue and UV emission intensities, respectively. Using the ratio of the blue and UV emissions, the LET_D_ to each OSLD in **(C)** is estimated through the calibration curve in **Figure 3C**. Finally, the intensity of the blue emissions in **(A)** is used to determine the dose to the OSLDs shown in **(D)** through the dose calibration in **Figure 3A**. Each LET determination in **(C)** has been used to correct the doses in **(D)** for ionization quenching through the correction factors derived from **Figure 3B**.

The readouts of the seven OSLDs in the DAPT fraction 2 are shown in **Figure 4**. The integral OSL emissions from each OSLD are shown in **Figures 4A, B** for the emissions in the blue and UV emission bands, respectively. To improve the OSL signal precision, each of the integral OSL emissions is scaled by the intensity of the reference irradiation as discussed in section 2.5. To determine the average LET_D_ to each OSLD, the ratio of the UV to its blue emission intensity is used to look-up the corresponding LET_D_ value in **Figure 3C**, where the determined LET_D_ values are shown in **Figure 4C**. The variation of determined LET_D_ values reflect the variation in the SOBP, where the highest LET_D_ occurs at the distal edge. The dose to each OSLD in **Figure 4D** is determined by applying the OSL dose calibration in **Figure 3A** to the blue emission readouts shown in **Figure 4A**, which are subject to ionization quenching. The determined LET_D_ values in **Figure 4C** are used to determine individual ionization quenching correction factors for each of the OSLDs in **Figure 4D** through the relative detector efficiency in **Figure 3B**.

### 3.2 Comparison of OSLD measurements and Monte Carlo

**Figure 5:**
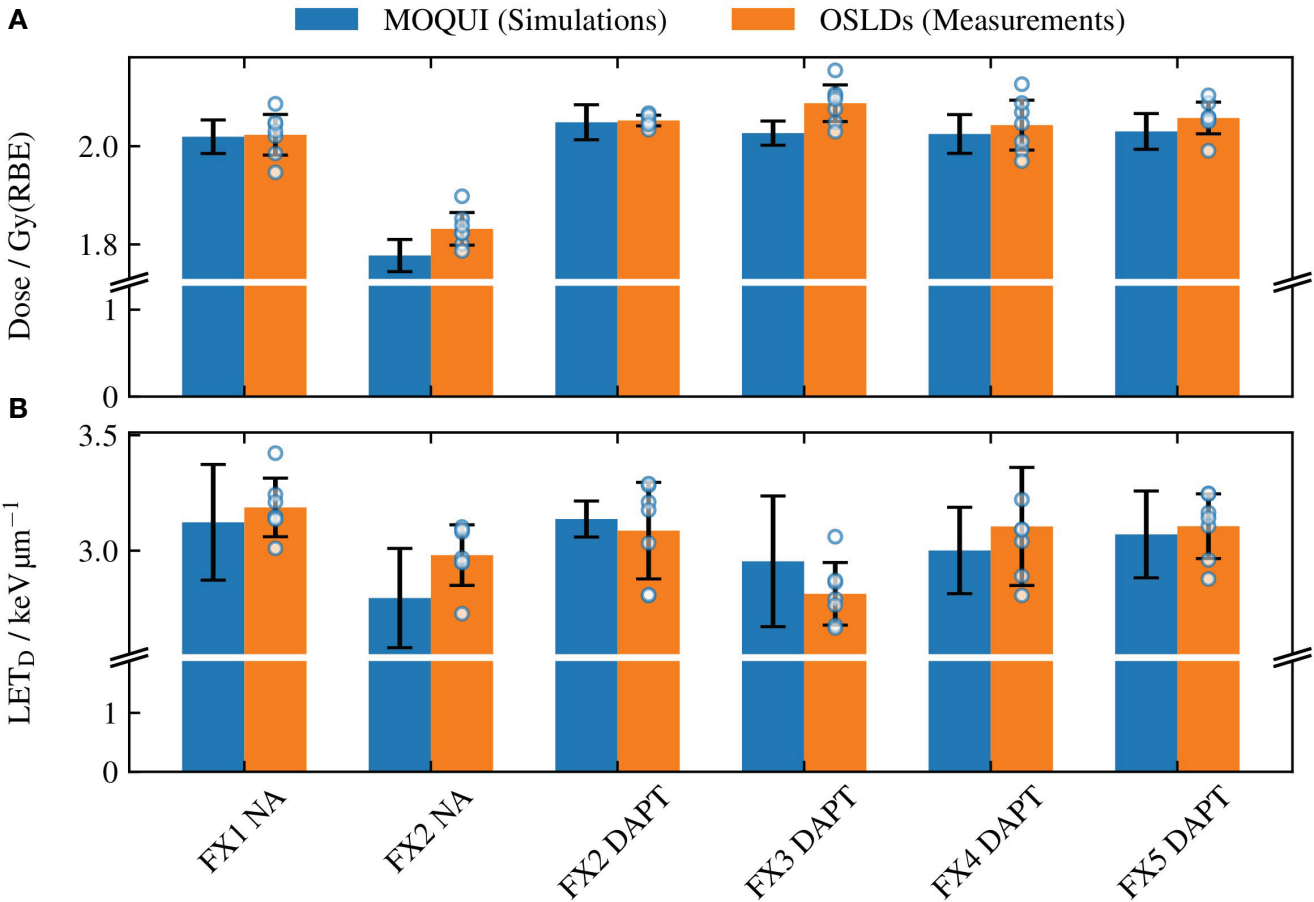
The mean **(A)** dose and **(B)** LET_D_ values determined through the Monte Carlo simulations with MOQUI and measurements with the OSLDs. For Monte Carlo, the uncertainty bars represent the standard deviation of the voxel-wise values acquired for each voxel within the OSLD contour. The uncertainty bars for the OSLDs show the standard deviation of the data of the seven OSLDs in each fraction. All uncertainties are given for a coverage factor *k=1*. The result from each OSLD is illustrated with a circular marker. The larger uncertainty bars in **(B)** relative to **(A)** are a result of the variations in LET_D_ throughout the circular OSLD arrangement (see **Figure 4**). The relative error between the simulated and measured values was 1.4% for the doses and 3.2% for the LET_D_. A significant loss of target coverage is observed for the NA delivery in fraction 2.

The results of the OSLD measurements and Monte Carlo simulations are summarized in **Figure 5** for different fractions for dose and LET_D_ in (**Figures 5A, B**) respectively. For the Monte Carlo results, the bar plots show the mean values obtained by averaging the voxel-wise values over the OSLD contour, whereas the uncertainty bars represent the standard deviation of the same values (coverage factor *k=1*). For the OSLD results, the bar plots show the same metrics obtained by averaging the measured values for the seven OSL detectors. In addition, the uncertainty bars for the OSLD measurements illustrate the standard deviation of the data. The overlay with circular markers shows the determined dose or LET_D_ from each OSLD measurement. As the LET_D_ varies throughout the delineated volume in the SOBP where the OSLDs are placed, there will be a spread of the experimental data. However, as the relative detector efficiency in **Figure 3B** varies only little with the LET_D_ for protons. This means that, e.g., a 10% deviation of the determined LET_D_ typically only leads to a 1% deviation of the LET_D_-derived quenching correction and consequently has a smaller impact on the accuracy of the dosimetry.

**Figure 6:**
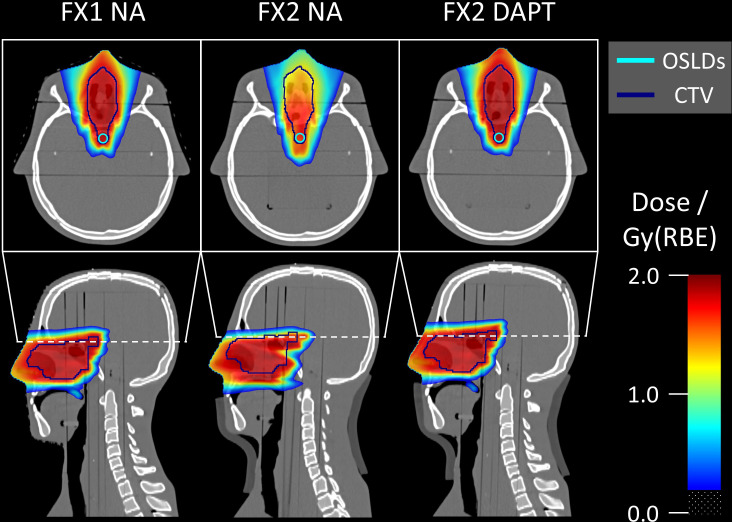
Monte Carlo simulated dose distributions superimposed on the CT scans. The single-slice OSLD contour used for Monte Carlo evaluation is visible on the axial slices. Fraction 1 represents the reference anatomy and positioning, matching the planning CT. Fraction 2 contains an offset and angle deviation caused by the introduced fat layer around the neck area. For fraction 2, DAPT considerably improves the dose conformity over NA. Doses below 0.2 Gy(RBE) are transparent in all slices.

**Figure 5** shows the NA delivery of the *reference plan* to fraction 1 (which replicates the planning CT) and the DAPT-generated *daily plans* delivered to the subsequent four fractions. In addition, the NA scenario for fraction 2 is shown as a worst-case example of the *reference plan* delivery among all fractions. For this fraction, the 1 cm fat layer around the phantom neck area caused a large spatial offset and angular deviation compared to the planning CT. The resulting loss of target coverage is further evident in **Figure 6**.

**Figure 7:**
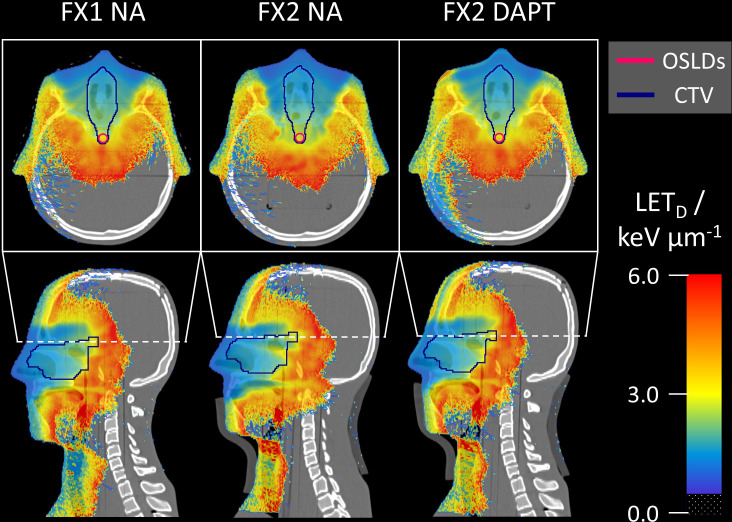
Monte Carlo simulated LET_D_ distributions superimposed on the same CT slices as in **Figure 6**. For fraction 2, NA shows higher LET_D_ values further downstream compared to DAPT, penetrating into the spinal cord. LET_D_ values below 0.5 keV/μm are transparent in all slices.

**Figure 6** shows the Monte Carlo dose distributions simulated for fractions 1 and 2. The dose distributions are superimposed on the axial and sagittal CT slices. FX1 NA represents the nominal *reference plan* delivered to the repositioned planning CT, while in FX2 NA the same plan is delivered to the mispositioned phantom (also including anatomical variations with the addition of a fat layer). The simulated underdose is clearly visible in both the axial and sagittal slices. Finally, DAPT restores the nominal dose conformity of FX1 NA by delivering the *daily plan* to fraction 2. Monte Carlo simulated LET_D_ distributions are shown on the same CT slices in **Figure 7**. Higher LET_D_ values are observed at the proton beam’s end-of-range. Due to the positioning and anatomical mismatch in fraction 2, these elevated LET_D_ values penetrate more deeply into the spinal cord for the NA delivery scenario.

## 4 Discussion

We demonstrated the first use of OSLDs for simultaneous dose and average LET determination in an anthropomorphic phantom for clinically plausible validation of a daily adaptive proton therapy treatment. These measurements allowed the verification of an online adaptive proton therapy workflow in terms of absolute dosimetry. The average LET was in this work only estimated as the LET_D_ for protons to demonstrate an applicability for RBE assessment, but could also have been estimated with respect to e.g. fluence-averaging. We simultaneously measured the point dose and LET_D_ in different geometries by varying the positioning and anatomy of a head-and-neck phantom. The Monte Carlo simulation results were in agreement with the OSLD measured values within uncertainties: The average relative error between the two was 1.4% and 3.2% for the doses and for the LET_D_, respectively. The good agreement between simulated and measured doses is a result of the high readout precision (< 1%) achievable with OSLDs, and the relatively low quenching correction factors (< 10%) estimated from the determined LET_D_. This is in contrast to OSLD dosimetry in carbon ion beams, where the quenching correction can exceed 50% and is the main contribution to the dose uncertainty with OSLDs (10).

The slightly larger relative LET_D_ deviation can result from several contributions. The determination of the LET_D_ relies on the UV/blue ratio from each OSLD, which is determined with a precision better than 0.6%, as the ratio of the two emission bands itself cancels out OSL material differences or sensitivity changes (7). Hence, the largest contribution to the LET_D_ determination uncertainty with OSLDs arises from the experimentally determined LET_D_ calibration curve in **Figure 3C**, where the (*k=1*) spread of the residuals is 1.8%, which is taken as an estimate of the systematic uncertainty. Due to the non-linearity of the LET_D_ calibration curve, the combined uncertainty depends on the LET_D_ value but is estimated to be around 2% (*k=1*). To improve the agreement between Monte Carlo simulations and experimental determination of the LET_D_, a more accurate LET_D_ calibration curve is needed.

Nevertheless, the use of OSLDs to determine the average LET enabled a correction of the ionization quenched response, which would not have been feasible with standard dosimetric methods such as e.g. radiochromic film or other solid-state detectors without an inherent way of estimating the averaged LET. This is particularly relevant in our case since we measured the delivery close to the beam’s end-of-range, as shown in **Figure 6**. While in the nominal/reference scenario the OSLDs are within the homogeneous region of the SOBP, a positioning error (such as in fraction 2) can lead to a displacement of the detectors into the dose fall-off region, where high dose and/or LET_D_ gradients can affect the measurements.

In addition, the novel experimental approach allowed us to verify underdosage in the target volume for delivery of the non-adapted *reference plan* to a modified phantom geometry (fraction 2). Applying the DAPT workflow to this fraction effectively restored the initially planned dose distribution, highlighting the efficacy of online adaptive proton therapy. As a result, our study measured and compared both adaptive and non-adaptive deliveries, a distinction from the previous experimental verification of DAPT (32), which measured only the adaptive delivery. Also, in that study, fractional changes were limited to adjustments in the nasal cavity fillings compared to the reference CT. In our study, DAPT was additionally tested for fractions with large offsets from the reference, verifying its applicability to more extreme cases. Another difference from the previous experiment was our use of OSLDs over radiochromic films, which allowed us to measure the absorbed dose rather than just a relative distribution.

While the use of OSLDs allowed for point-like measurements, seven OSLDs were employed to map the dose and LET_D_ in the ROI. For radiation fields with sharp dose or LET_D_ gradients, this presents a challenge due to the averaging of the signal over the 3-mm diameter OSLD surface. In our specific case, this was a greater concern for LET_D_ than for dose measurements due to the high LET gradients within the ROI (**Figures 6**, **7**). For this reason, our circular OSLD arrangement would ideally be replaced by high-resolution measurements with an OSLD film. Further investigations are planned to extend the dose and LET_D_ determination from point to 2D measurements. Furthermore, the OSLD readout protocol used a two-week delay between irradiation and readout to minimize the effect of signal fading. For OSLDs to be used effectively in daily QA, modifications to this readout procedure are essential. For the same reason, real-time use of OSLDs is not feasible during online adaptive proton therapy treatments, where changes in patient anatomy and tumor motion can occur in the time frame of minutes to seconds (12). However, other clinical applications could potentially be envisioned for *in vivo* patient measurements, such as cases with cardiac devices or pregnant patients.

Limitations of our study include that the implementation of the PSI *Gantry 2* beam model in MOQUI was only cross-validated with the treatment planning system and was never independently verified through direct measurements. In addition, there are inherent uncertainties in the delineation of the OSLD ROI contour on the planning CT, as well as image registration uncertainties for the contours propagated from the planning CT to the daily fraction images. Despite these aspects, there was a notable agreement between the Monte Carlo evaluation and both measured values of dose and LET_D_, highlighting the robustness of our methodology.

In conclusion, we found the OSLDs to be a suitable choice for the simultaneous measurement of the absolute point dose and LET_D_ delivered by multi-field proton irradiation to an anthropomorphic phantom. This innovative approach allowed us to validate the efficacy of an online adaptive proton therapy workflow under clinically relevant conditions. The fact that OSLD detectors can be cut to almost any size and shape makes them perfectly suited for in-phantom measurements within a predefined ROI. While our focus was the experimental validation study of an online adaptive proton therapy workflow, the same approach could be applied to a variety of clinical innovation projects and workflow developments that require accurate measurements of the point dose and LET.

## Data availability statement

The raw data supporting the conclusions of this article will be made available by the authors, without undue reservation.

## Author contributions

MB: Conceptualization, Data curation, Formal analysis, Investigation, Methodology, Resources, Software, Validation, Visualization, Writing – original draft, Writing – review & editing. JC: Conceptualization, Data curation, Formal analysis, Investigation, Methodology, Resources, Software, Validation, Visualization, Writing – original draft, Writing – review & editing. HL: Methodology, Resources, Software, Validation, Writing – review & editing. EC: Investigation, Methodology, Writing – review & editing. KC: Funding acquisition, Investigation, Methodology, Writing – review & editing. MT: Methodology, Resources, Writing – review & editing. SS: Writing – review & editing, Resources, Software. EY: Funding acquisition, Methodology, Supervision, Writing – review & editing. BW: Methodology, Supervision, Writing – review & editing. AL: Funding acquisition, Methodology, Resources, Supervision, Writing – review & editing. HP: Funding acquisition, Methodology, Supervision, Writing – review & editing. FA: Funding acquisition, Methodology, Resources, Supervision, Writing – review & editing. KN: Conceptualization, Data curation, Formal analysis, Funding acquisition, Methodology, Resources, Supervision, Validation, Writing – original draft, Writing – review & editing.

## References

[1] Paganetti H Blakely E Carabe-Fernandez A Carlson DJ Das IJ Dong L. Report of the AAPM TG-256 on the relative biological effectiveness of proton beams in radiation therapy. Med Phys (2019) 46:e53–78. doi: 10.1002/mp.13390 PMC9559855 30661238

[2] Grün R Friedrich T Traneus E Scholz M. Is the dose-averaged LET a reliable predictor for the relative biological effectiveness? Med Phys (2019) 46:1064–74. doi: 10.1002/mp.13347 30565705

[3] Underwood TSA McNamara AL Appelt A Haviland JS Sørensen BS Troost EGC. A systematic review of clinical studies on variable proton Relative Biological Effectiveness (RBE). Radiotherapy Oncol (2022) 175:79–92. doi: 10.1016/j.radonc.2022.08.014 35988776

[4] Angellier G Gautier M Hérault J. Radiochromic EBT2 film dosimetry for low-energy protontherapy. Med Phys (2011) 38:6171–7. doi: 10.1118/1.3654161 22047382

[5] Vana N Schöner W Fugger M Akatov Y. Absorbed dose measurement and LET determination with TLDs in space. Radiat Prot Dosimetry (1996) 66:145–52. doi: 10.1093/oxfordjournals.rpd.a031703

[6] Sawakuchi GO Sahoo N Gasparian P Rodriguez M Archambault L Titt U. Determination of average LET of therapeutic proton beams using Al_2_O_3_:C optically stimulated luminescence (OSL) detectors. Phys Med Biol (2010) 55:4963–76. doi: 10.1088/0031-9155/55/17/006 20693613

[7] Christensen JB Togno M Bossin L Pakari OV Safai S Yukihara EG. Improved simultaneous LET and dose measurements in proton therapy. Sci Rep (2022) 12:8262. doi: 10.1038/s41598-022-10575-4 35585205 PMC9117334

[8] Granville D Sahoo N Sawakuchi GO. Calibration of the Al_2_O_3_:C optically stimulated luminescence (OSL) signal for linear energy transfer (LET) measurements in therapeutic proton beams. Phys Med Biol (2014) 59:4295–310. doi: 10.1088/0031-9155/59/15/4295 25029434

[9] Yukihara EG Doull BA Ahmed M Brons S Tessonnier T Jäkel O. Time-resolved optically stimulated luminescence of Al_2_O_3_:C for ion beam therapy dosimetry. Phys Med Biol (2015) 60:6613–38. doi: 10.1088/0031-9155/60/17/6613 26270884

[10] Christensen JB Muñoz ID Bassler N Stengl C Bossin L Togno M. Optically stimulated luminescence detectors for dosimetry and LET measurements in light ion beams. Phys Med Biol (2023) 68:155001–1. doi: 10.1088/1361-6560/acdfb0 37336242

[11] Paganetti H Botas P Sharp GC Winey BA. Adaptive proton therapy. Phys Med Biol (2021) 66. doi: 10.1088/1361-6560/ac344f PMC8628198 34710858

[12] Albertini F Matter M Nenoff L Zhang Y Lomax AJ. Online daily adaptive proton therapy. Br J Radiol (2020) 93:20190594. doi: 10.1259/bjr.20190594. 31647313 PMC7066958

[13] Qiu Z Olberg S den Hertog D Ajdari A Bortfeld T Pursley J. Online adaptive planning methods for intensity-modulated radiotherapy. Phys Med Biol (2023) 68:10TR01–1. doi: 10.1088/1361-6560/accdb2 PMC10637515 37068488

[14] Kurz C Nijhuis R Reiner M Ganswindt U Thieke C Belka C. Feasibility of automated proton therapy plan adaptation for head and neck tumors using cone beam CT images. Radiat Oncol (2016) 11. doi: 10.1186/s13014-016-0641-7 PMC4851791 27129305

[15] Jagt T Breedveld S van de Water S B Heijmen M. Hoogeman. Near real-time automated dose restoration in IMPT to compensate for daily tissue density variations in prostate cancer. Phys Med Biol (2017) 62:4254–72. doi: 10.1088/1361-6560/aa5c12 28140380

[16] Moriya S Tachibana H Hotta K Nakamura N Sakae T Akimoto T. Range optimization for target and organs at risk in dynamic adaptive passive scattering proton beam therapy – A proof of concept. Physica Med (2018) 56:66–73. doi: 10.1016/j.ejmp.2018.11.010 30527091

[17] Bernatowicz K Geets X Barragan A Janssens G Souris K Sterpin E. Feasibility of online IMPT adaptation using fast, automatic and robust dose restoration. Phys Med Biol (2018) 63:085018. doi: 10.1088/1361-6560/aaba8c 29595145

[18] Botas P Kim J Winey BA Paganetti H. Online adaption approaches for intensity modulated proton therapy for head and neck patients based on cone beam CTs and Monte Carlo simulations. Phys Med Biol (2018) 64:015004. doi: 10.1088/1361-6560/aaf30b 30524097

[19] Jagt T Breedveld S van Haveren R Heijmen B Hoogeman M. An automated planning strategy for near real-time adaptive proton therapy in prostate cancer. Phys Med Biol (2018) 63:135017–7. doi: 10.1088/1361-6560/aacaa7 29873296

[20] Jagt T Breedveld S van Haveren R Nout RA Astreinidou E Heijmen B. Plan-library supported automated replanning for online-adaptive intensity-modulated proton therapy of cervical cancer. Acta Oncol (2019) 58:1440–5. doi: 10.1080/0284186x.2019.1627414 31271076

[21] Matter M Nenoff L Meier G Weber DC Lomax AJ Albertini F. Intensity modulated proton therapy plan generation in under ten seconds. Acta Oncol (2019) 58:1435–9. doi: 10.1080/0284186x.2019.1630753 31271095

[22] Nenoff L Matter M Hedlund Lindmar J Weber DC Lomax AJ Albertini F. Daily adaptive proton therapy – the key to innovative planning approaches for paranasal cancer treatments. Acta Oncol (2019) 58:1423–8. doi: 10.1080/0284186x.2019.1641217 31364904

[23] Matter M Nenoff L Marc L Weber DC Lomax AJ Albertini F. Update on yesterday’s dose—Use of delivery log-files for daily adaptive proton therapy (DAPT). Phys Med Biol (2020) 65:195011. doi: 10.1088/1361-6560/ab9f5e 32575083

[24] Nenoff L Matter M Jarhall AG Winterhalter C Gorgisyan J Josipovic M. Daily adaptive proton therapy: is it appropriate to use analytical dose calculations for plan adaption? Int J Radiat OncologyBiologyPhysics (2020) 107:747–55. doi: 10.1016/j.ijrobp.2020.03.036 32275996

[25] Bobić M Lalonde A Sharp GC Grassberger C Verburg JM Winey BA. Comparison of weekly and daily online adaptation for head and neck intensity-modulated proton therapy. Phys Med Biol (2021) 66:055023. doi: 10.1088/1361-6560/abe050 PMC8313628 33503592

[26] Lalonde A Bobić M Winey BA Verburg JM Sharp GC Paganetti H. Anatomic changes in head and neck intensity-modulated proton therapy: Comparison between robust optimization and online adaptation. Radiotherapy Oncol (2021) 159:39–47. doi: 10.1016/j.radonc.2021.03.008 PMC8205952 33741469

[27] Nesteruk KP Bobić M Lalonde A Winey BA Lomax AJ Paganetti H. CT-on-Rails versus in-Room CBCT for Online Daily Adaptive Proton Therapy of Head-and-Neck Cancers. Cancers (2021) 13:5991. doi: 10.3390/cancers13235991 34885100 PMC8656713

[28] Nesteruk KP Bobić M Sharp GC Lalonde A Winey BA Nenoff L. Low-dose computed tomography scanning protocols for online adaptive proton therapy of head-and-neck cancers. Cancers (2022) 14:5155. doi: 10.3390/cancers14205155 36291939 PMC9600085

[29] Bobić M Lalonde A Nesteruk KP Lee H Nenoff L Gorissen BL. Large anatomical changes in head-and-neck cancers – A dosimetric comparison of online and offline adaptive proton therapy. Clin Trans Radiat Oncol (2023) 40:100625–5. doi: 10.1016/j.ctro.2023.100625 PMC10120292 37090849

[30] Lalonde A Bobić M Sharp GC Chamseddine I Winey BA Paganetti H. Evaluating the effect of setup uncertainty reduction and adaptation to geometric changes on normal tissue complication probability using online adaptive head and neck intensity modulated proton therapy. Phys Med Biol (2023) 68:115018–8. doi: 10.1088/1361-6560/acd433 PMC10351361 37164020

[31] Pedroni E Bearpark R Böhringer T Coray A Duppich J Forss S. The PSI Gantry 2: a second generation proton scanning gantry. Z für Medizinische Physik (2004) 14:25–34. doi: 10.1078/0939-3889-00194 15104007

[32] Nenoff L Matter M Charmillot M Krier S Uher K Weber DC. Experimental validation of daily adaptive proton therapy. Phys Med Biol (2021) 66:205010. doi: 10.1088/1361-6560/ac2b84 34587589

[33] Safai S Bula C Meer D Pedroni E. Improving the precision and performance of proton pencil beam scanning. Trans Cancer Res (2012) 1:196–206. doi: 10.21037/599

[34] Marks LB Yorke ED Jackson A Ten Haken RK Constine LS Eisbruch A. The use of normal tissue complication probability (NTCP) models in the clinic. Int J Radiat oncology biology Phys (2010) 76:S10–9. doi: 10.1016/j.ijrobp.2009.07.1754 PMC4041542 20171502

[35] Ahmed MF Eller SA Schnell E Ahmad S Akselrod MS Hanson OD. Development of a 2D dosimetry system based on the optically stimulated luminescence of Al2O3. Radiat Measurements (2014) 71:187–92. doi: 10.1016/j.radmeas.2014.01.009

[36] Yukihara EG McKeever SWS. Spectroscopy and optically stimulated luminescence of Al2O3:C using time-resolved measurements. J Appl Phys (2006) 100. doi: 10.1063/1.2357344

[37] Christensen JB Togno M Nesteruk KP Psoroulas S Meer D Weber DC. Al_2_O_3_:C optically stimulated luminescence dosimeters (OSLDs) for ultra-high dose rate proton dosimetry. Phys Med Biol (2021) 66:085003. doi: 10.1088/1361-6560/abe554 33571973

[38] Yukihara EG McKeever SWS Andersen CE Bos AJJ Bailiff IK Yoshimura EM. Luminescence dosimetry. Nat Rev Methods Primers (2022) 2. doi: 10.1038/s43586-022-00102-0

[39] Yukihara EG Christensen JB Togno M. Demonstration of an optically stimulated luminescence (OSL) material with reduced quenching for proton therapy dosimetry: MgB4O7:Ce,Li. Radiat Measurements (2022) 152:106721. doi: 10.1016/j.radmeas.2022.106721

[40] Nascimento LF Leblans P van der Heyden B Akselrod MS Goossens J Verellen D. Characterization and quenching correction for a 2D real time radioluminescent system in therapeutic proton and carbon charged beams. Sensors Actuators A: Phys (2022) 345:113781–1. doi: 10.1016/j.sna.2022.113781 PMC9737660 36501879

[41] Gambarini G Artuso E Camoni G Colombo G Felisi M Gebbia A. Let quenching correction in solid state dosimeters. Physica Med (2016) 32:245–5. doi: 10.1016/j.ejmp.2016.07.517

[42] Lee H Shin J Verburg JM Bobić M Winey BA Schuemann J. MOQUI: an open-source GPU-based Monte Carlo code for proton dose calculation with efficient data structure. Phys Med Biol (2022) 67:174001. doi: 10.1088/1361-6560/ac8716 PMC9513828 35926482

[43] Grassberger C Trofimov A Lomax AJ Paganetti H. Variations in linear energy transfer within clinical proton therapy fields and the potential for biological treatment planning. Int J Radiat Oncol Biol Phys (2011) 80:1559–66. doi: 10.1016/j.ijrobp.2010.10.027 PMC3094592 21163588

